# Robust Stabilization Control Based on Guardian Maps Theory for a Longitudinal Model of Hypersonic Vehicle

**DOI:** 10.1155/2014/270172

**Published:** 2014-03-25

**Authors:** Yanbin Liu, Mengying Liu, Peihua Sun

**Affiliations:** College of Astronautics, Nanjing University of Aeronautics and Astronautics, Nanjing 210016, China

## Abstract

A typical model of hypersonic vehicle has the complicated dynamics such as the unstable states, the nonminimum phases, and the strong coupling input-output relations. As a result, designing a robust stabilization controller is essential to implement the anticipated tasks. This paper presents a robust stabilization controller based on the guardian maps theory for hypersonic vehicle. First, the guardian maps theories are provided to explain the constraint relations between the open subsets of complex plane and the eigenvalues of the state matrix of closed-loop control system. Then, a general control structure in relation to the guardian maps theories is proposed to achieve the respected design demands. Furthermore, the robust stabilization control law depending on the given general control structure is designed for the longitudinal model of hypersonic vehicle. Finally, a simulation example is provided to verify the effectiveness of the proposed methods.

## 1. Introduction

With the development of advanced flight control technologies, the resulting demands become higher due to the complicated and diverse flight tasks. Also, some new design challenges in combination with the uncertain flight conditions and the unknown internal dynamics will have a tremendous impact on the conventional design means of flight control [1]. As a result, the design ideas of flight control are required to constantly update to satisfy the complex performance requirements. In the past decades, the flight control technologies rapidly develop with the help of the mathematical tools, the manufacturing means, and the computer simulations [2]. Typically, some traditional design ways such as the root locus method, the eigenvalue assignment method, and the Bode analysis method are used to obtain the control parameters related to the according tasks [3]. However, if the control structure becomes more and more complicated and associated with the large flight range, these control design means depending on the given trim point may become insufficient because the realization of the multiple demands of flight stability is difficult in consideration of the different trim states, especially the presence of some bizarre flight points due to the design errors corresponding to the control parameters. These may make overall stability unable to be satisfied throughout the whole flight envelope [4–6].

In order to enhance flight stability and manipulating capability, some advanced control strategies are introduced in the design process of flight control. These design methods consist of the robust adaptive control [7], the sliding mode control [8], the dynamic inversion control [9], the fuzzy control [10], the neural control [11], and so on. Nevertheless, the flight control laws in terms of these modern control methods need to acquire the feedback of the complete flight states which are difficult to measure in the practical application [12–15]. On the other hand, these proposed controllers are limited to use due to some unrealistic assumptions and harsh prerequisites [16]. For example, the dynamic inversion control can relieve the coupling relations corresponding to the coupling states by building the affine linearization model [17]. Besides that, such resulting model is more accurate for the control design in comparison to the linear model acquired by Taylor's series expansion; the control robustness is improved accordingly [18]. Unfortunately, the dynamic inversion control relies too much on the airplane model, and in some case the affine linearization model is difficult to obtain due to the strong coupling dynamics between the inputs and the outputs. In addition, the dynamic inversion control is necessary to connect with the other control methods so as to improve system robustness and control adaptability [19]. In general, the conventional flight control approaches cannot guarantee overall stability; the control structures and parameters need to be changed with regard to the different trim conditions. The advanced flight control methods depend on the established model, providing the entire feedback states in the control design process. For this reason, new control methods should be introduced to design the satisfactory control law for the modern aerospace vehicle.

Theoretically, the realization of the flight control system is based on the vehicle models gotten by small perturbation linearization or linear variable parameter transformation [20]. As soon as the vehicle model is built, the control law can be designed by means of the linear interpolation or the application of linear parameter varying (LPV) control structure [21]. Although the linear interpolation is commonly used in practice, the stability issues in the control boundaries are difficult to be addressed. Alternatively, the LPV control is too conservative for implementing the stabilization control over the large flight range. On the other hand, the LPV control needs to solve the matrix inequalities which might be singular in some flight points [22]. As a result, the new control ideas need to be introduced to guarantee generalized stability, whereas the robust stabilization control based on the guardian maps can establish the constraint relations between the open subsets of complex plane and the eigenvalues of the state matrix of closed-loop control system [23]. Accordingly, the control parameters corresponding to the fixed control structure can be automatically regulated by assigning the poles of the closed-loop control system in accordance with the anticipated stability region [24].

As for hypersonic vehicle, the overall structure is special to implement the challenging tasks under the complicated flight conditions corresponding to the high altitude and speed [25]. To this end, the vehicle model exhibits the unconventional dynamic characteristics such as the unstable poles and the rigid-elastic coupling mode [26]. In addition, some new challenges need to be faced for hypersonic flight, including the trajectory optimization [27], the scramjet propulsion design [28], and the multidisciplinary integrated iteration [29]. More importantly, the hypersonic vehicle is easily affected by the external disturbances, the unknown model dynamics, and the uncertain coupling actions, leading to worsening of the anticipated performances due to the deviation of the waveriding design points. As a result, the robust stabilization controller designed is critical for hypersonic vehicle to maintain the continuous and stable flight states. Based on that, this paper puts forward the robust stabilization control methods using the guardian maps theories for a longitudinal model of hypersonic vehicle. Also, a scheduling algorithm of the control gains is applied for this hypersonic vehicle model, while adopting the state matrix in relation to the needed parameters determined by the guardian map restrains. Furthermore, a simulation example is performed to verify the effectiveness of the proposed control methods.

## 2. Matrix Polynomial Family and Guardian Maps Theory

In principle, the generalized stability with regard to the matrix polynomial is characterized by all system poles located in the left half-plane. However, if some design goals such as the bandwidth, damping, and response time are considered, the design area must be specifically defined to meet the respected handling qualities. According to these stability demands, the resulting stability region can be set using the guardian maps defined as follows.

**Definition 1 (see [30])**

Let  *χ*  be the set of all  *n* × *n*  square matrices, or the set of all polynomials of degree at most* n*, and let  *S*  be an open subset of  *χ*. Let  *ν*  map  *χ*  into the complex field C. One thinks that  *ν*  guards  *S*  if for all$\;x\in \overset{-}{S}$, the equivalence

(1) $$\begin{array}{c} \nu \left(x\right)=0⟺x\in \partial S \end{array}$$

holds. In this situation, one says that  *ν*  is a guardian map for *S*  when  ∂*S* denotes the border of  *S*. Based on (1), one further assumes that  *x*(*r*_0_) ∈ *S*  for some  *r*_0_ ∈ *U* then has [30]

(2) $$\begin{array}{c} \forall r\in U,\;x\left(r\right)\in S⟺\forall r\in U,\;\nu \left(x\left(r\right)\right)\neq 0. \end{array}$$

Equation (2) demonstrates that the stability issue can be replaced by guarded stability sets using the guardian map. Also, we find that this guardian map  *ν*(*x*(*r*))  is the function of  *r*, and in this case  *x*(*r*)  is stable with regard to the given region  *Ω*  only if (1)  *x*(*r*_0_)  is stable corresponding to  *Ω*; (2)  *ν*(*x*(*r*))  has no zeros along with  [*r*_−_, *r*_+_]  , where  *r*_−_,  *r*_+_  are, respectively, the minimum and maximum of  *r*. In other words, if  *ν*  constrains  *x*(*r*)  and  *x*(*r*_0_) ∈ *Ω*,  *x*(*r*)  is stable with regard to *Ω* only if  *ν*(*x*(*r*))  is not equal to zero in the interval of  [*r*_−_, *r*_+_]  [24]. As a result, we need to identify whether  *x*(*r*)  maintains stable along with  *Ω*, so the related work is to check whether  *ν*[*A*(*r*)]  exists zeros for the interval  [*r*_−_, *r*_+_]  such that testing stability problem is convenient for the control design. Accordingly, the guardian maps are provided for some typical regions as follows [23].

(1) The left half-plane region  *χ*_1_  with regard to  *Re*(*z*) < *α*  is guarded by

(3) $$\begin{array}{c} v_{\alpha }\left(\chi_{1}\right)=\det\left({\chi_{1}}^{2}⊙I-\alpha I⊙I\right)\det\left(\chi_{1}-\alpha I\right), \end{array}$$

where  ⊙  represents the bialternate product.

(2) The conic area  *χ*_2_  with inner angle  2*θ*  is guarded by

(4) νξ(χ2)=det⁡(χ22⊙I+(1−2ξ2)χ2⊙χ2)det⁡(χ2)ξ=cos⁡θ.

(3) The circle sector  *χ*_3_  of radius  *ω* > 0  is guarded by

(5) νω(χ3)=det⁡(χ3⊙χ3−ω2I⊙I) ·det⁡(χ3−ωI)det⁡(χ3+ωI).

Furthermore, the resulting sector  *χ*_1_∩*χ*_2_∩*χ*_3_  is guarded by [12]

(6) $$\begin{array}{c} \nu \left(\chi_{1}\cap \chi_{2}\cap \chi_{3}\right)=\nu_{\alpha }\left(\chi_{1}\right)\nu_{\xi }\left(\chi_{2}\right)\nu_{\omega }\left(\chi_{3}\right). \end{array}$$

**Figure 1:**
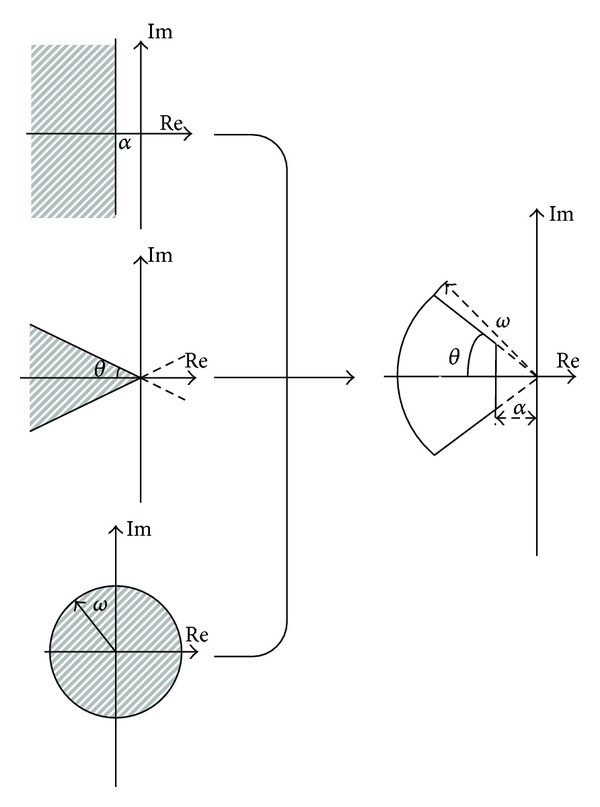
Construction sector in terms of classic stability section.

According to (6), we can construct new domains from the classic region to satisfy the real design demands, and this stability area is demonstrated in Figure 1. In addition, the guardian maps have other intrinsic transformation properties with regard to symmetry, translation, and scaling [30]. Using these characteristics, the guardian maps equations corresponding to some new sections can be obtained accordingly, such that other design requirements with consideration of the anticipated goals can be realized.

## 3. General Control Law Design Based on Guardian Maps Theory

**Figure 2:**
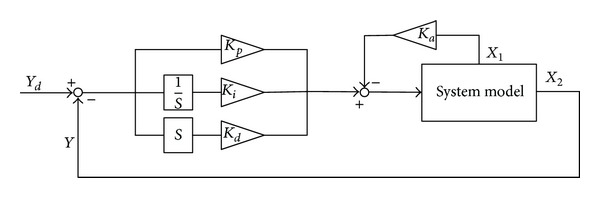
General structure of control system.

For the given system model, this paper applies the classical control structure to realize the stability and track control. It is expressed in Figure 2.

In Figure 2, as soon as the initial control gains  *K*_*a*_,  *K*_*p*_,  *K*_*i*_,  *K*_*d*_  are given, the according poles of the closed system lie in the region  *Ω* = *Ω*(*α*, *ξ*, *ω*)  shown in Figure 1. Correspondingly, this closed system can be written in the following form:

(7) $$\begin{array}{c} A\left(r\right)=A_{0}+rA_{1}+\cdots +r^{k}A_{k}, \end{array}$$

where  *A*  denotes the state matrix of this closed system. Therefore, the resulting guardian map  *v*_*Ω*_[*A*(*r*)]  is only relevant to the control gain matrix  *r*, composed of  *K*_*a*_, *K*_*p*_, *K*_*i*_, *K*_*d*_, and the anticipated region can be designed as *Ω*_*t*_ = *Ω*(*α*_*t*_, *ξ*_*t*_, *ω*_*t*_)  where in  *α*_*t*_,  *ξ*_*t*_,  *ω*_*t*_  are required to meet the performance indices, so the design goal corresponding to the control action is to make that  *Ω* = *Ω*(*α*, *ξ*, *ω*)  can approach to *Ω*_*t*_ = *Ω*(*α*_*t*_, *ξ*_*t*_, *ω*_*t*_)  by constantly iterating the control gains  *K*_*a*_,  *K*_*p*_,  *K*_*i*_,  *K*_*d*_. In each iterative process, the according region in Figure 1 is used if all eigenvalues defined as  Λ = {*λ*_1_, *λ*_2_,…, *λ*_*n*_} lie in the left half plane. In this case, the corresponding region  *Ω*_Λ_ = *Ω*(*α*_Λ_, *ξ*_Λ_, *ω*_Λ_) determined by Λ = {*λ*_1_, *λ*_2_,…, *λ*_*n*_}  will be compared with the respected goal region. Therefore, the integrated region is obtained as follows [24]:

(8) αU=max⁡{αt,αΛ}ξU=min⁡{ξt,ξΛ}ωU=max⁡{ωt,ωΛ},

where

(9) αΛ=max⁡{Re(λi)}ξΛ=min⁡{ξ(λi)}ωΛ=max⁡{|λi|}.

**Figure 3:**
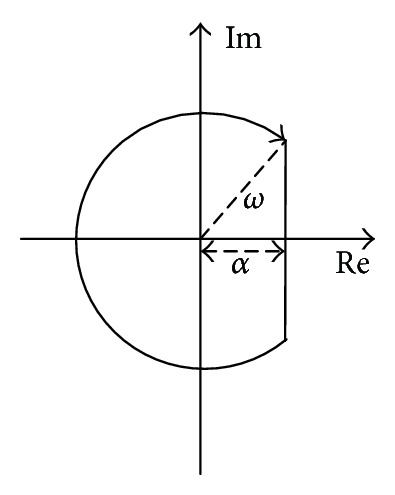
Construction sector in combination with instability region.

In particular, if any pole among  Λ = {*λ*_1_, *λ*_2_,…, *λ*_*n*_}  is located in the right half plane, the resulting region is redefined as in Figure 3.

According to Figure 3, the resulting sector can be depicted by

(10) αU=αΛωU=max⁡{ωt,ωΛ},

where

(11) αΛ=max⁡{Re(λi)}ωΛ=max⁡{|λi|}.

Based on (8) and (10), the according sector is gotten with regard to each iteration process that does not stop until this resulting area completely matches with the anticipated region. In addition to the selection of the iterative region, how to change the control gain matrix is equally important. Following that, we assume the control parameters  *r*_0_  can make that the eigenvalues belong to the iterative region decided by (8) and (10). At this time, we can obtain the interval range corresponding to  *r*  by solving  *v*_*Ω*_[*A*(*r*)]. They are expressed by [23]

(12) $$\begin{array}{c} r^{-}\approx \sup\left\{r<r_{0}:v_{\Omega }\left[A\left(r\right)\right]=0\right\}\left(\text{or}-\infty \;\text{if}\;\text{none}\;\text{exists}\right)\\ r^{+}\approx \inf\left\{r<r_{0}:v_{\Omega }\left[A\left(r\right)\right]=0\right\}\left(\text{or}-\infty \;\text{if}\;\text{none}\;\text{exists}\right). \end{array}$$

To regulate the control gain matrix  *r* = [*K*_*a*_, *K*_*p*_, *K*_*i*_, *K*_*d*_], first we select  *K*_*a*_  as the variable, whereas other parameters are fixed, and in this case calculate  *r* ^−^  and  *r* ^+^ for  *Ω*_Λ_ = *Ω*(*α*_Λ_, *ξ*_Λ_, *ω*_Λ_)  to get *K*_*a*max⁡_  and  *K*_*a*min⁡_. Following that,  *K*_*a*_  is assigned to  (*K*_*a*max⁡_ + *K*_*a*min⁡_)/2, and then the similar works are performed for each control gain such that the control gain matrix is continually updated as  *r*′ = [*K*_*a*_′, *K*_*p*_′, *K*_*i*_′, *K*_*d*_′]. Thus, new design region  *Ω*_Λ_  is identified with  *r*′ = [*K*_*a*_′, *K*_*p*_′, *K*_*i*_′, *K*_*d*_′]. Furthermore, this iterative process does not stop until  *Ω*_Λ_ ⊂ *Ω*_*t*_.

## 4. Modeling and Flight Control Design for Hypersonic Vehicle

The dynamic models of hypersonic vehicle tend to be highly complex and multidisciplinary due to strong interaction between aerodynamics, propulsion, structure, and controls [31]. Not only that, the special configuration applied to design hypersonic vehicle makes that the established model is unstable, nonminimum phase, and strong coupling relations. These will bring new challenges for the control system design, as well as the model establishment. Based on the Lagrange equation, the longitudinal model of hypersonic vehicle is built by [31]

(13) V˙=1m(Tcos⁡α−D)−gsin(θ−α)α˙=1mV(−Tsinα−L)+q+gVcos⁡(θ−α)q˙=MIyyh˙=Vsin(θ−α)θ˙=q,

where  *V*,  *h*,  *α*,  *θ*,  *q*  represent the flight velocity, the flight altitude, the angle of attack, the pitch angle, and the pitch angle change rate of hypersonic vehicle, respectively;  *L*,  *D*,  *M*  denote, respectively, the lift, the drag, and the pitch moment;  *m*,  *I*_*yy*_  indicate the vehicle mass and the moment of inertia, respectively. The aerodynamic forces and moments of the nonlinear model in (13) need to be acquired to build the complete relations between the systems inputs and outputs. They are expressed by

(14) L≈12ρV2SCL(α,δe)D≈12ρV2SCD(α,δe)M≈12ρV2Sc−[CM,α(α)+CM,δe(δe)],

where  *ρ*  represents air density;  *S*,$\;\;\overset{-}{c}\;$ denotes the reference area and the mean aerodynamic chord, respectively. Additionally, the oriented control model established is critical to design the flight control system. In this paper, the aerodynamic coefficients with regard to the oriented control model are selected as the following polynomials [18]:

(15) CL(α,δe)=CLαα+CLδeδe+CL0CD(α,δe)=CDα2α2+CDαα+CDδe2δe2+CDδeδe+CD0CM,α=CM,αα2α2+CM,ααα+CM,α0CM,δe=ceδe.

Besides these aerodynamic forces and moments, this paper considers the thrust expression as [18]

(16) CT=cΦΦΦ¨=−2ξϖΦ˙−ϖ2Φ+ϖ2Φc,

where  Φ  denotes the fuel to air ratio, whereas  Φ_*c*_  indicates the propulsive ratio command. In this study, the elevator deflection angles  *δ*_*e*_  and  Φ_*c*_  are selected as the control inputs to stabilize and follow the according commands. In particular, applying the approximate polynomials to depict the aerodynamic coefficients may bring some modeling errors, but these expressions are more suitable for designing the control law, as well as analyzing the dynamic characteristics. Therefore, in order to obtain these model polynomials of hypersonic vehicle, the corresponding identification methods need to be used to estimate these polynomial coefficients. For example, the trust region method can be adopted to identify these polynomial coefficients [32]. The core idea of this method is to first define an adjacent area with regard to the current iteration point  *x*_*k*_, and this area is called the trust region written by

(17) $$\begin{array}{c} \Pi_{k}=\left\{x\;|\;||x-x_{k}||\leq h_{k}\right\}, \end{array}$$

where  *h*_*k*_  denotes the upper bound of step. Furthermore, we adopt the trust region method to get the according coefficients in (15). For the lift expression, the criterion function is provided by

(18) $$\begin{array}{c} G\left(K\right)=\frac{1}{2}{\left|C_{L}\left(K,\bar{X}\right)-\bar{Y}\right|}^{2}, \end{array}$$

where  *K*  is the coefficients matrix  *K*_*L*_ = [*C*_*L*_^*α*^, *C*_*L*_^*δ*_*e*_^, *C*_*L*_^0^];$\;\bar{X}\;$is the matrix constituted of the given flight state datum, whereas$\;\bar{Y}\;$represents the given lift coefficients.$\;\bar{X}\;$and$\;\bar{Y}\;$can be acquired by means of the parametric modeling and computational fluid dynamics methods. Moreover, the tentative mean is used to implement the iteration process, and then we have [32]

(19) s=xi+1−ximin⁡x F=min⁡x{12sTHfs+sTgi}||Did||≤hi,

where  *s* indicates the iterative step;  *H*_*f*_  is the selected symmetric matrix that is obtained by

(20) Hf=[∂2F∂x12⋯∂2F∂x1∂xn⋮⋱⋮∂2F∂xn∂x1⋯∂2F∂xn2]gi=grad(F)=∇(F)=(∂F∂x1,⋯∂F∂xn).

Once the expression  *H*_*f*_ + *λI*  is positive definite matrix when  *λ* ≥ 0, (20) is solvable. In this case, we have

(21) $$\begin{array}{c} ||{\left(H_{f}+\lambda I\right)}^{-1}s||=h. \end{array}$$

Further, we can set the next step to perform the iterative process, and lastly the optimal results can be obtained for the lift coefficients matrix  *K*_*L*_. Similarly, the polynomial coefficients with regard to  *C*_*D*_, *C*_*M*_  can be gotten accordingly; thus these aerodynamic expressions are introduced to construct the nonlinear model. For the further application in the control design, this model needs to be simplified. Commonly, there are two means to complete the model simplification. One is to obtain the linear model by the small perturbation linearization, and the other is to transform the nonlinear model to the equivalent model, such as the feedback linearized model or the linear varying parameter model. This paper utilizes the linear model to design the control system for hypersonic vehicle. It is expressed by [33]

(22) ΔX˙=A·ΔX+B·ΔUΔX=[ΔV,Δα,Δq,Δh,Δθ]TΔU=[ΔΦ,Δδe]T,

where

(23) A=[XvXα0Xh−gZvVT0ZαVT01−ZqVT0ZhVT00MvMαMqMh00−V000V000100]B=[XδeXΦZδeVT0ZΦVT0MδeMΦ0000],

where  *X*,  *Z*,  *M*  indicate the coefficients of the linear model. For (22), the characteristic polynomial regarding the short-term mode is written by

(24) $$\begin{array}{c} \Delta_{sp}=V_{t}s^{2}-\left(Z_{\alpha }+V_{t}M_{q}\right)s+M_{q}Z_{\alpha }-V_{t}M_{\alpha }. \end{array}$$

The according solutions of (24) are gotten by

(25) $$\begin{array}{c} s=\frac{Z_{\alpha }}{2V_{t}}\pm \frac{\sqrt{{\left(Z_{\alpha }-V_{t}M_{q}\right)}^{2}+4V_{t}^{2}M_{\alpha }}}{2V_{t}}. \end{array}$$

Under normal circumstances, hypersonic vehicle is designed as the special slim construction such that the aerodynamic forces suffered from the forebody section are large enough in comparison to the other parts. Such configuration makes that the aerodynamic focus is prior to the gravity centre, namely,  *M*_*α*_ > 0. For (25), there exists the positive root, and this means the short-term motion is unstable for hypersonic vehicle. Furthermore, when removing the long term parts in (23), we have

(26) [s−ZαV0−1+ZqV0−Mαs−Mq][Δα(s)Δq(s)] =[ZϕV0MϕZδeV0Mδe]T[Δϕ(s)Δδe(s)].

According to (26), the frequency and damping corresponding to the short-term mode are estimated by

(27) ωsp≈ZαV0Mq−(1+ZqV0)Mαζsp≈(−Zα/V0−Mq)(2ωsp).

Based on that, we see that the angle of attack and pitch angle change rate will affect the short-term mode. Thus, if these flight states can be introduced into the control inputs, the system performances will be ameliorated accordingly. Additionally, we can design the augmentation control loop in line with the structure in Figure 2. In this case, only  *K*_*aα*_,  *K*_*aq*_  need to be adjusted and tuned. Alternatively, the control structure can be adopted for the pitch angle control loop, the altitude control loop, and the velocity control loop by applying the general control structure in Figure 2. Especially, the according control laws based on Figure 2 can be provided as follows:

(28) Δδea=KaαΔα+KaqΔqΔδe=Δδea+[Kpθ(Δθd−Δθ)+Kiθ∫(Δθd−Δθ)]Δθd=KahΔh˙+Kph(Δhd−Δh)+Kih∫(Δhd−Δh)ΔΦ=KpV(ΔVd−ΔV)+KiV∫(ΔVd−ΔV),

where  *K*_*aα*_,  *K*_*aq*_  denote the augmentation control parameters;  *K*_*pθ*_,  *K*_*iθ*_  are the pitch angle control gains;  *K*_*ah*_,  *K*_*ph*_,  *K*_*ih*_  represent the altitude control gains;  *K*_*pV*_,  *K*_*iV*_  indicate the speed control gains. The control block diagram of hypersonic vehicle is demonstrated in Figure 5.

**Figure 4:**
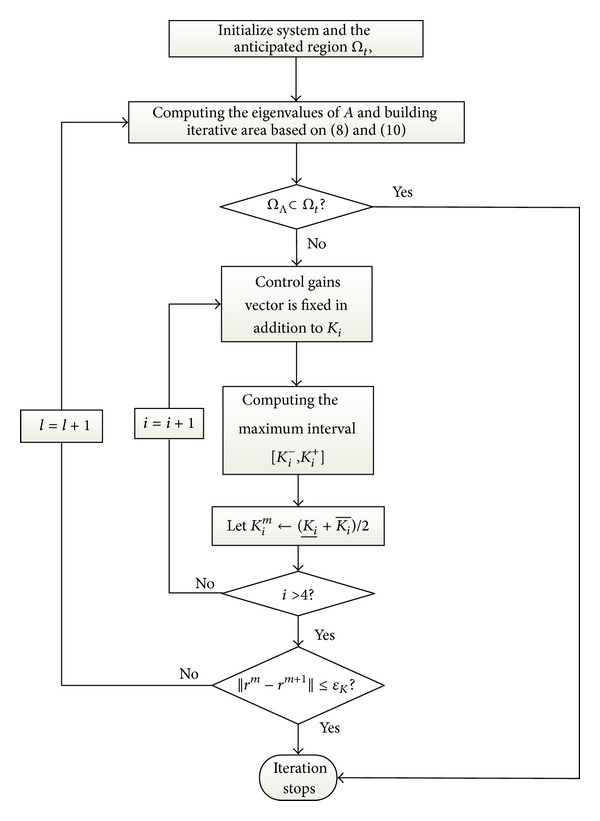
Iteration flowchart with regard to control gains.

**Figure 5:**
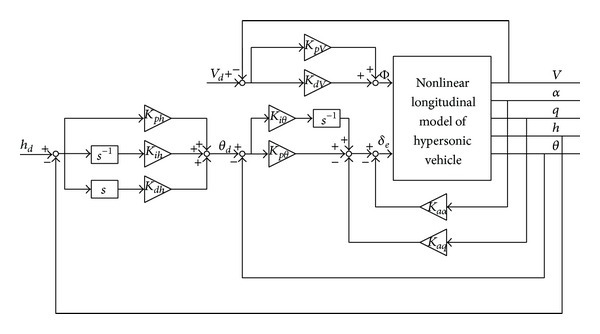
Control block diagram of hypersonic vehicle.

From Figure 5, we note that the control block diagram is constituted of the general control structure in Figure 2, and the according control parameters can be obtained based on the iteration process in Figure 4. With the combination of the inner loop and the outer loop, the complete control configuration is formed to achieve the track control responses with regard to the altitude and the velocity.

## 5. Simulation Example and Analysis

To verify the effectiveness of the control system based on the guardian maps theories, this paper applies the hypersonic vehicle model in [26], and the aerodynamic coefficients of this model are provided by

(29) CL=0.6203αCD=0.6450α2+0.0043378α+0.003772CM=−0.035α2+0.036617α+5.3261×10−6+0.0292(δe−α)CT=0.02576[1−164(α−αtrim)2]β,

where  *α*_trim_  is the trim angle of attack of this hypersonic vehicle model. Also, the model properties are used in [34]. By introducing these aerodynamic parameters to the nonlinear model, the trim states with regard to  *V*_0_ = 4500 m/s,  *h*_0_ = 33500 m  include  *α*_trim_ = 2.845°,  *δ*_*e*trim_ = −0.564°,  *β*_*c*trim_ = 0.217. The resulting characteristic roots of this model are gotten as − 0.737,  0.623,  −0.0000663 ± 0.0366102*j*  wherein there exists one positive root that demonstrates that this model is unstable. Additionally, the characteristic roots with regard to the long-term mode are close to the imaginary axis, and this shows the long-term motion is underdamped. As a result, the control action is crucial to guarantee flight stability as well as to realize the command track.

**Figure 6:**
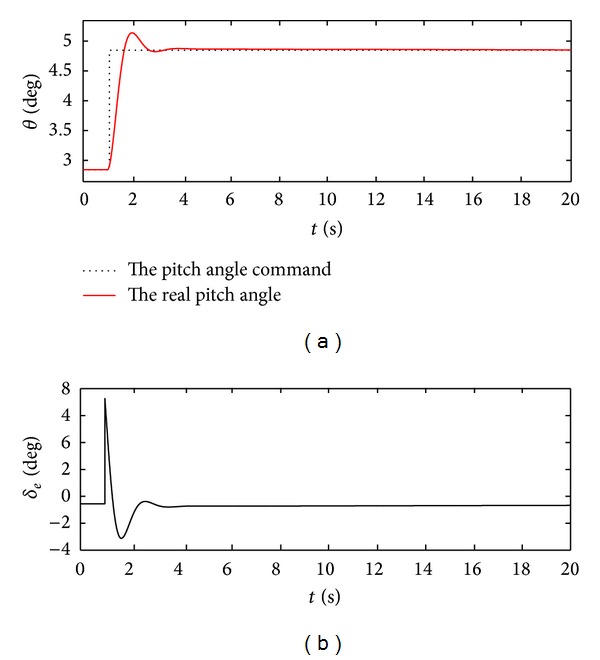
Pitch angle response and elevator deflection angle.

Because this model has the unstable and nonminimum phase characteristics, first we need to design the stability augmentation system to improve system performances. Based on the control structure in Figure 2, the guardian maps theories are applied to acquire the stability augmentation gain  *K*_*aα*_. Beyond this, we select the design region in consideration of the system instability in Figure 3, including  *α*_*tα*_ = 0.01,  *ω*_*tα*_ = 10. By the continuous iteration, the augmentation parameter is gotten as  *K*_*aα*_ = 4.0368  such that the characteristic roots lie in the left-half plane or the imaginary axis. Furthermore, the performance qualities with regard to the short-term mode are considered, and they consist of  *α*_*ts*_ = −1,  *ω*_*ts*_ = 10,  *ξ*_*ts*_ = 0.35  which are satisfied with the flight qualities demands [35]. In this situation, the control gain of the pitch angle rate is gotten as  *K*_*aq*_ = 2.0687, and the resulting characteristic roots of the short-term mode are − 3.37 ± 1.2*i*. After that, the quality requirements with respect to the long-term mode are given as  *α*_*tl*_ = −0.02,  *ω*_*tl*_ = 5,  *ξ*_*tl*_ = 0.1, and accordingly the control gains are obtained as  *K*_*pθ*_ = 3.9245,  *K*_*iθ*_ = 13.1620  such that the resulting characteristic roots of the long-term mode are − 0.0200 ± 0.0300*i*. Based on these acquired control parameters, the track simulation can be done to test the validity of the attitude controller. Correspondingly, the response curves to the step signal  Δ*θ*_*d*_ = 2 deg⁡  at the 2 second are shown in Figure 6.

According to Figure 6, we find that the pitch angle can rapidly follow the attitude command signal, and the elevator deflection angle reaches the resulting trim value as the system enters into the new stability states. This shows that the proposed control law can guarantee system stability and track ability even if the vehicle model is unstable. Compared with other control methods, the structure of this controller is simple, and the control gains are adaptively obtained in terms of the anticipated control qualities such that the controller is feasible to apply for the real design.

**Figure 7:**
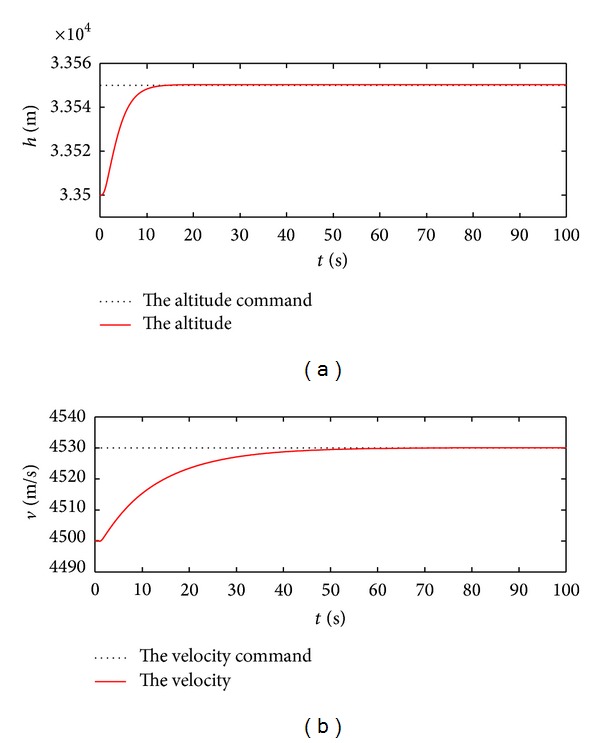
Response curves to the altitude and velocity commands.

**Figure 8:**
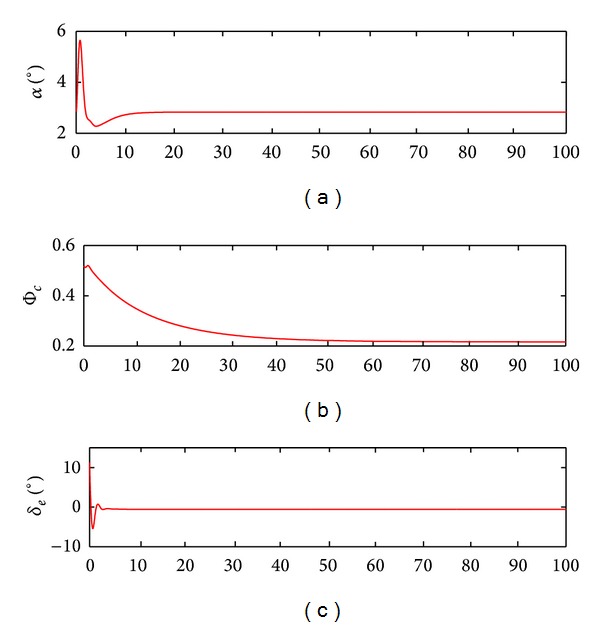
Change curves of the control inputs and angle of attack.

Furthermore, the altitude and velocity control gains can be gotten based on the similar iteration process in line with the guardian maps theories, and these gains are  *K*_*ph*_ = 0.001,  *K*_*dh*_ = 0.004 and *K*_*pV*_ = 0.01,  *K*_*dV*_ = 0.005. In the following simulation, the commands are  Δ*h*_*d*_ = 50 m,  Δ*V*_*d*_ = 30 m/s, and the resulting response curves after 100 seconds are demonstrated in Figures 7 and 8.

**Figure 9:**
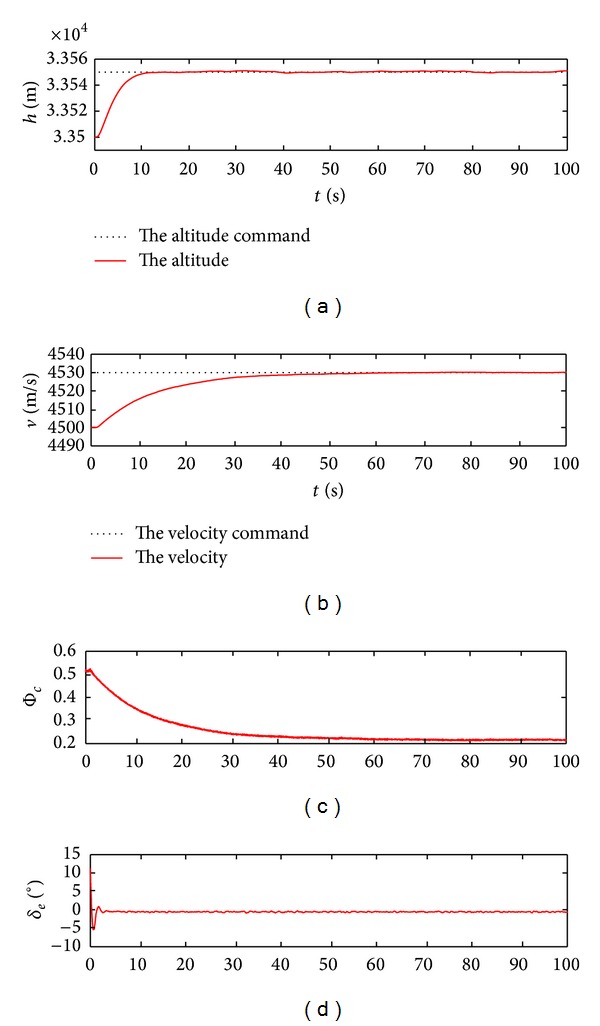
Change curves of the control inputs and angle of attack.

According to Figures 7 and 8, the real altitude and velocity can track the respective command signals well. This manifests that the control action relieves the coupling relations between the inputs and the outputs, while ensuring that the flight states and control inputs rapidly reach the new steady values without the presence of the undesirable oscillation. On the further consideration of system robustness, the model uncertainties including 50% errors concerning the lift and drag coefficients are exerted in the simulation. The according response curves are acquired in Figure 9.

Figure 9 tells us that the system outputs can correspond to the given commands even in the presence of the large model uncertainties. Although there are some flutters in the response process, the track errors are small and the changes of the control inputs are smooth. This shows that the control actions are effective to restrain the uncertain disturbances and provide strong system robustness. More importantly, the control gains are obtained automatically by means of the adaptive iteration methods in terms of the given goals. Such design methods based on the guardian maps theories make that the overall system satisfies the anticipated quality demands, thus resulting in suppressing the unstable model dynamics and the uncertain effects.

## 6. Conclusion

This paper proposes the robust stabilization control methods based on the guardian maps theories for a longitudinal model of hypersonic vehicle. There are three aspects that need to be considered for the control law design. The first part is to build the general control structure to realize the iteration process of the control gains in line with the guardian maps theories. The second issue is to establish the control-oriented model using the system identification methods, and at the same time the linear model can be acquired according to the small perturbation linearization principles. The last problem is to design the robust stabilization controller for the unstable model of hypersonic vehicle in combination with the general control configuration and the presented iterative process. We believe this work is helpful to design the complicated robust control law and implement the adaptive gain adjustment for hypersonic vehicle in the future.
